# Novel Method to Flag Cardiac Implantable Device Infections by Integrating Text Mining With Structured Data in the Veterans Health Administration’s Electronic Medical Record

**DOI:** 10.1001/jamanetworkopen.2020.12264

**Published:** 2020-09-21

**Authors:** Hillary J. Mull, Kelly L. Stolzmann, Marlena H. Shin, Emily Kalver, Marin L. Schweizer, Westyn Branch-Elliman

**Affiliations:** 1 Center for Healthcare Organization and Implementation Research (CHOIR), VA Boston Healthcare System, Boston, Massachusetts; 2 Department of Surgery, Boston University School of Medicine, Boston, Massachusetts; 3 Center for Access and Delivery Research and Evaluation, Iowa City VA Health Care System, Iowa City, Iowa; 4 Department of Internal Medicine, University of Iowa, Iowa City; 5 Department of Medicine, VA Boston Healthcare System, Boston, Massachusetts; 6 Harvard Medical School, Boston, Massachusetts

## Abstract

**Importance:**

Health care–associated infections (HAIs) are preventable, harmful, and costly; however, few resources are dedicated to infection surveillance of nonsurgical procedures, particularly cardiovascular implantable electronic device (CIED) procedures.

**Objective:**

To develop a method that includes text mining of electronic clinical notes to reliably and efficiently measure HAIs for CIED procedures.

**Design, Setting, and Participants:**

In this multicenter, national cohort study using electronic medical record data for patients undergoing CIED procedures in Veterans Health Administration (VA) facilities for fiscal years (FYs) 2016 and 2017, an algorithm to flag cases with a true CIED-related infection based on structured (eg, microbiology orders, vital signs) and free text diagnostic and therapeutic data (eg, procedure notes, discharge summaries, microbiology results) was developed and validated. Procedure data were divided into development and validation data sets. Criterion validity (ie, positive predictive validity [PPV], sensitivity, and specificity) was assessed via criterion-standard manual medical record review.

**Exposures:**

CIED procedure.

**Main Outcomes and Measures:**

The concordance between medical record review and the study algorithm with respect to the presence or absence of a CIED infection. CIED infection in the algorithm included 90-day mortality, congestive heart failure and nonmetastatic tumor comorbidities, CIED or surgical site infection *International Statistical Classification of Diseases and Related Health Problems, Tenth Revision, Clinical Modification *(*ICD-10-CM*) diagnosis codes, antibiotic treatment of *Staphylococci*, a microbiology test of a cardiac specimen, and text documentation of infection in specific clinical notes (eg, cardiology, infectious diseases, inpatient discharge summaries).

**Results:**

The algorithm sample consisted of 19 212 CIED procedures; 15 077 patients (78.5%) were White individuals, 1487 (15.5%) were African American; 18 766 (97.7%) were men. The mean (SD) age in our sample was 71.8 (10.6) years. The infection detection threshold of predicted probability was set to greater than 0.10 and the algorithm flagged 276 of 9606 (2.9%) cases in the development data set (9606 procedures); PPV in this group was 41.4% (95% CI, 31.6%-51.8%). In the validation set (9606 procedures), at predicted probability 0.10 or more the algorithm PPV was 43.5% (95% CI, 37.1%-50.2%), and overall sensitivity and specificity were 94.4% (95% CI, 88.2%-97.9%) and 48.8% (95% CI, 42.6%-55.1%), respectively.

**Conclusions and Relevance:**

The findings of this study suggest that the method of combining structured and text data in VA electronic medical records can be used to expand infection surveillance beyond traditional boundaries to include outpatient and procedural areas.

## Key Points

**Question:**

How can coded and free-text data from electronic medical records be used to support infection tracking and other patient safety surveillance following common but understudied cardiac device procedures?

**Findings:**

In this national cohort study of 19 212 patients who underwent cardiovascular implantable electronic device procedures in the US Department of Veterans Affairs health care system, an algorithm to reliably identify cases with a true 90-day infection by combining coded data (eg, diagnosis of a comorbid condition) and free-text data extracted from clinical notes (eg, documentation of an infection by a cardiologist) was developed and validated. Text note searching was a useful and straightforward adjunct to coded data for surveillance.

**Meaning:**

The findings of this study suggest that the algorithm to detect patients who received cardiovascular implantable electronic device and developed an infection has the potential to significantly enhance surveillance in an underserved area.

## Teaser

This cohort study describes the development of an algorithm to identify cardiac implantable device infections using coded and free-text data from the Veterans Health Administration’s electronic medical record.

## Introduction

Health care–associated infections (HAIs) are among the top 10 causes of death in the US, accounting for more than 99 000 deaths and more than $10 billion in treatment and care costs annually.^1,2,3,4,5^ Infection prevention programs, including HAI surveillance with infection detection and feedback to clinicians and administrators, can markedly reduce infections, health care costs, and improve patient safety.^6,7,8^ HAI surveillance can also be used on an ongoing basis to assess the effectiveness of already-implemented prevention strategies. Although HAI surveillance for performance measurement and quality improvement is routine for surgical procedures and inpatient care,^9^ a gap remains in HAI surveillance for outpatient and procedural settings, including the cardiac electrophysiology laboratory.

Detection of HAIs, particularly surgical site infections (SSIs) associated with invasive outpatient or nonsurgical procedures, is rare because there are no infection reporting mandates directing resources toward surveillance. Recently, the US Centers for Disease Control and Prevention’s National Healthcare Safety Network (NSHN) initiated a voluntary surveillance program for SSIs in nontraditional settings, including cardiac catherization laboratories.^10,11^ The program requires surveillance through manual record review because, as with other types of postprocedure complications, diagnosis codes produce inaccurate and incomplete results.^12,13,14^ Prior attempts to create electronic SSI detection tools to augment or replace the NHSN manual review process perform poorly; however, these tools were limited to structured data fields in electronic medical records (EMRs; eg, microbiology order or diagnosis code) and missed essential information recorded only in free-text form in clinical notes.^15,16,17^ A successful automated infection monitoring system may benefit from augmentation with clinical note data.

Thus, we sought to develop a method to detect procedures with a high likelihood of a true HAI that integrates structured EMR data with text mining of electronic clinical notes. Our approach has the potential to enhance case ascertainment and reduce manual medical record review burden. We focused on infections following cardiovascular implantable electronic device (CIED) procedures using data from the Veterans Health Administration (VA). CIED infection rates vary from 1% to 5% and most require readmission and reoperation; mortality from deep CIED infections approaches 19%, and each infection is estimated to cost more than $50 000, placing a high premium on prevention.^18,19,20^ Measurement is key to designing effective quality improvement processes to prevent HAI^21^; however, inadequate infection prevention resources are available in the electrophysiology laboratory, creating a critical need for surveillance data.^22,23,24^ Previously, diagnosis codes and medical record review of a random sample of CIED procedures were used to detect postprocedure infections, but both approaches had low positive predictive validity (PPV).^25,26^

We developed and tested a predictive algorithm based on coded EMR data that improved PPV but overestimated the true rate of infection compared with medical record review data.^26^ With the rich electronic clinical data (including note text) available from the VA EMR and a large national, longitudinal sample, we developed and validated a novel approach to expanding HAI surveillance.

## Methods

Our novel method of developing and validating an HAI algorithm used retrospective data from the VA and data analysis tools in SQL Server Studio 17 (Microsoft). Criterion validity of the algorithm (ie, PPV, sensitivity, and specificity) was assessed with criterion-standard manual medical record review.^27^ The VA Boston Healthcare System institutional review board approved this study; informed consent was waived because database data were used. This study followed the Strengthening the Reporting of Observational Studies in Epidemiology (STROBE) reporting guideline for cohort studies.^28^

### Data Sources and Study Population

All CIED procedures (eg, pacemaker or cardiac defibrillator implantation) performed between October 2016 and September 2017 were sampled, and cases were evenly divided into development and validation data sets. Diagnostic and therapeutic data were pulled from the VA Corporate Data Warehouse’s (CDW) administrative files (ie, visit and hospital stay dates, diagnoses, and procedure codes), structured tables (laboratory orders and results, pharmacy orders and dispensed name, quantity and fill dates, vital signs), and text files (clinical notes containing unstructured text organized by date and type).^29^ Patient characteristics (ie, age, sex, race, ethnicity, and marital status) and diagnostic history according to the Agency for Healthcare Research and Quality’s Comorbidity Software were also extracted. Criterion validity was assessed by criterion standard manual medical record review using the Joint Legacy Viewer interface (US Department of Defense and VA).^30^

### Definition of CIED Infection

Our algorithm outcome measure was any 90-day CIED infection, assessed by applying standard definitions (eTable 1 in the Supplement).^31,32,33^ CIED infections were defined as device pocket infections, lead infections, and/or endocarditis. Infection status was determined either from physician documentation of infection, or at least 2 of the following criteria: documented presence of symptoms (eg, fever), positive laboratory tests, or the initiation of antibiotic treatment up to 90 days postprocedure. CIED infections present at the time of procedure, superficial cellulitis in other locations, and stitch abscesses were not recorded as CIED infections.^34^

### Algorithm Development

We used an iterative approach to develop an algorithm to predict CIED procedures with a true infection. We began with previously developed clinical diagnostic and treatment flags from structured data^26^: temperature at or higher than 100.3 °F (38.0 °C), any antibiotic prescribed for more than 3 days, antibiotics typically used to treat *Staphylococcal* infections, microbiology test order, and an *International Statistical Classification of Diseases and Related Health Problems, Tenth Revision, Clinical Modification* (*ICD-10-CM*) code diagnosis of either an SSI, nonspecific infection, or cardiac device infection (eTable 2 in the Supplement). Next, we considered other factors that might affect the presence of infection-related flags, including comorbidities, emergent procedure, and death within 90 days. Then, we examined data in text-based fields from the clinical notes or microbiology results using a broad list of keyword strings, including *pacemaker* and *infection* (eTable 3 in the Supplement). We created flags for documentation of a postprocedure infection, documentation of a CIED infection prior to the procedure, a potentially relevant microbiology result with *Staphylococcus aureus* organism, or a microbiology result with coagulase negative *Staphylococci*.

We randomly sampled cases flagged with infection codes and/or microbiology orders in stages to direct manual review and identify ways to improve flags. Examples of potential adjustments included altering keywords for text note searching (eg, include or exclude the word *cellulitis*) and limiting the types of notes that keyword searches were applied to (eg, excluding instructions about how to prevent cardiac device infection and when to return to the hospital). We also randomly sampled and reviewed 50 cases with no structured or text-based flags. Our medical record review process followed accepted guidelines including a standardized review tool.^35^ Two reviewers (M.S. and E.K.) were trained on 5 cases and then reviewed an additional 5 cases at a time until they reached an interrater reliability (IRR) κ score greater than 90%.^36^ The lead clinician on the study (W.B.E.) performed training, served as the criterion standard for IRR measurement, rereviewed 15 cases during the study, and answered questions. Once reliability was established, reviewers worked separately and were masked to flagged data.

We used the manually reviewed cases to develop an algorithm to assign probability of infection using a logistic regression model controlling for fixed effects and adjusted for nonnormal predictors (PROC GLIMMIX [SAS Institute]).^37^ We included the various CIED infection flags as well as patient comorbidities and demographic information. We then iteratively tested combinations of structured and text-data flags and covariates until our algorithm reached a sensitivity of greater than 70%. We used the results from the development round to set a threshold of predicted probability of infection that could be used to direct manual investigation of the case.

### Algorithm Validation

We applied the final version of the algorithm to our validation sample to obtain predicted probabilities that a CIED device infection occurred within a 90-day window after an electrophysiology procedure. Based on the predicted probability threshold selected at the development stage, we randomly selected 80% of CIED cases that met the threshold for medical record review. We also reviewed a random sample of 1% of the CIED cases with predicted probability that did not meet the threshold to ensure that the algorithm was not missing a high proportion of true infection cases. We assessed the criterion validity of the final iteration of the algorithm in the medical record–reviewed validation sample and compared the C statistics, areas under the receiver operating characteristic curve (AUCs), and receiver operating characteristic (ROC) curves of the development and validation logistic regression models.

### Statistical Analysis

Our analyses included bivariate comparisons with infection outcomes (χ^2^ test and *t* tests as appropriate) in the development data set. We also calculated odds ratios for infection for our predictive model and calculated the AUC and ROC curve for the final algorithm in the development and then validation data sets. We performed a sensitivity analysis to ensure results did not change because of the 1 VA facility that does not enter clinical notes into CDW and comparing algorithm performance. All analyses were completed using SAS software version 9.2 (SAS Institute). The threshold for statistical significance was set to *P* < .05 in 2-tailed tests.

## Results

### Patient Sample

We identified 19 212 CIED procedures from FY 2016 to 2017. Most patients were White race (15 077 [78.5%]) and male sex (18 766 [97.7%]). Race and sex data were missing in 895 patients (4.7%) and 66 patients (0.3%), respectively. The mean (SD) age in our sample was 71.8 (10.6) years. No age data were missing. Characteristics were similar in the development and validation samples.

### Algorithm Development

The algorithm development sample included half of all CIED procedures in FYs 2016 to 2017: 9606 procedures performed in 67 VA medical centers. Table 1 shows the patient, procedure. and postprocedure characteristics of the development sample; details on patient comorbidities are available from the authors. To develop and refine the version of the algorithm created in the first stage of development, we completed 3 rounds of medical record review of the cases flagged and unflagged by keyword search (381) and identified 47 infections (12.3%). We achieved an IRR κ score of 94% after 1 round of training medical record review.

**Table 1.** Patient and Procedure Characteristics in FY 2016-2017 Development Sample, Medical Record Review Sample, and Cases With Infection

Variable	No. (%)		
	Flag rate in FY 2016-2017 development sample (N = 9606)	Medical record reviewed	
		Total (n = 381)	Infection (n = 47)
**Procedure outcomes**			
Medical record review confirmed infection	NA	47 (12.34)	47 (100)
Died <90 d postprocedure	260 (2.7)	30 (7.87)	7 (14.89)
**Patient characteristics**			
Age, mean (SD), y	71.98 (10.58)	72.03 (10.45)	70.63 (9.50)
Race			
White	7537 (78.46)	295 (77.43)	43 (91.49)^a^
African American	1487 (15.48)	64 (16.8)	2 (4.26)^a^
Race not identified or other^b^	582 (6.06)	22 (5.77)	2 (4.26)
Hispanic ethnicity	520 (5.41)	25 (6.56)	3 (6.38)
Female	207 (2.15)	9 (2.36)	1 (2.13)
Select comorbidities			
Congestive heart failure	4872 (50.72)	230 (60.37)	22 (46.81)^a^
Coagulopathy	548 (5.7)	38 (9.97)	8 (17.02)
Diabetes			
Without chronic complications	3870 (40.29)	187 (49.08)	23 (48.94)
With chronic complications	2661 (27.7)	146 (38.32)	17 (36.17)
Obesity	1991 (20.73)	102 (26.77)	10 (21.28)
Pulmonary circulation disease	412 (4.29)	25 (6.56)	3 (6.38)
Renal failure	2463 (25.64)	123 (32.28)	14 (29.79)
Solid tumor without metastasis	792 (8.24)	34 (8.92)	7 (14.89)
**Characteristics of potential infection**^**c**^			
Billing			
Emergent problem	1193 (12.42)	55 (14.44)	10 (21.28)
*ICD-10-CM* code^d^			
CIED infection	362 (3.77)	110 (28.87)	37 (78.72)^e^
SSI infection only	53 (0.55)	11 (2.89)	4 (8.51)^a^
Unspecified infection	130 (1.35)	16 (4.2)	2 (4.26)
Vitals			
Body temperature ≥38 °C up to 30 d postprocedure	260 (2.71)	42 (11.02)	7 (14.89)
Pharmacy order			
No antibiotic ordered	7726 (80.43)	162 (42.52)	15 (31.91)
Any antibiotics or drug class with duration ≥3 consecutive d, ordered between 6-90 d postprocedure	1880 (19.57)	219 (57.48)	32 (68.09)
Antibiotic			
Typically used to treat to *Staphylococci*	1572 (16.36)	185 (48.56)	30 (63.83)^a^
Not typically used to treat *Staphylococci*	308 (3.21)	34 (8.92)	2 (4.26)
Laboratory orders			
Blood specimens from bacteriology reports with positive cultures that had antibiotic susceptibility testing performed	906 (9.43)	162 (42.52)	41 (87.23)^e^
Cardiac specimens from bacteriology reports with positive cultures that had antibiotic susceptibility testing performed	160 (1.67)	48 (12.6)	21 (44.68)^e^
Miscellaneous (eg, wound, abscess) specimens from bacteriology reports with positive cultures that had antibiotic susceptibility testing	923 (9.61)	49 (12.86)	1 (2.13)^a^
Laboratory results			
Microbiology order and keyword search of microbiology results found *Staphylococcus aureus* organism	56 (0.58)	17 (4.46)	7 (14.89)^f^
Microbiology order and keyword search of microbiology results found coagulase negative *Staphylococcal* species organism	43 (0.45)	15 (3.94)	4 (8.51)
Microbiology order, but keyword search of microbiology results found no evidence of *Staphylococci* flag	1791 (18.64)	195 (51.18)	32 (68.09)^a^
Clinical note text			
Keyword search for *infection* from 3-90 d postprocedure, select note titles	750 (7.81)	226 (59.32)	45 (95.74)^e^
Keyword search for *infection* (0/1) 3-90 d preprocedure, select note titles	357 (3.72)	81 (21.26)	10 (21.28)

Abbreviations: CIED, cardiovascular implantable electronic device; FY, fiscal year; *ICD-10-CM*, *International Statistical Classification of Diseases, Tenth Revision, Clinical Modification*; NA, not applicable; SSI, surgical site infections.

^a^ *P* < .05.

^b^ Other includes American Indian or Alaska Native, Asian, Native Hawaiian or other Pacific Islander, and multiracial.

^c^ Procedure characteristics used as flags in infection detection algorithm.

^d^ Detailed lists of antibiotics, *ICD-10-CM* codes, and text keywords are available in eTable 2 in the Supplement.

^e^ *P* < .001.

^f^ *P* < .01.

In comparing individual text-based flags with medical record review results, we observed that clinical note type and keyword combinations to detect postprocedure infection performed well, while text searches to rule out present-on-admission or unrelated infections (eg, pneumonia) were not useful and unlikely to improve the predictive value of the algorithm. Similarly, using 1 or 2 spaces between word combinations, accounting for spelling fluctuations, and preserving word order (eg, *CIED* + *infection*) yielded better results when compared with keyword searches with more liberal allowances (eg, 5 words between *CIED* and *infection*).

The final infection flagging algorithm included 90-day mortality, the comorbidities congestive heart failure and solid tumor without metastasis, an *ICD-10-CM* CIED or SSI infection diagnostic code, antibiotics typically used to treat *Staphylococci*, and an order of a cardiac microbiology specimen. Two flags based on keywords documented in cardiology clinical notes were also significant: a positive flag for keywords related to infection diagnosis (eg, *pocket infection*, *endocarditis*) and a positive flag for keywords related to cardiac device infection recorded in preprocedure clinical notes as a negative predictor of postoperative infection (Table 2). Excluding the VA facility without text data did not produce a significantly different algorithm. Notably, the SSI code flag included both SSI and wound dehiscence codes, per the NSHN guideline, yet only wound dehiscence codes were documented in our CIED data.

**Table 2.** CIED Infection Detection Algorithm Logistic Regression Results for Development and Validation

Variables	OR (95%CI)	
	Development sample	Validation sample
CIED procedures, No.	381	363
Infections, No.	47	107
Procedure outcomes		
Died <90 d postprocedure	15.24 (2.3-100.84)	1.87 (0.57-6.17)
Comorbidity		
Congestive heart failure	0.13 (0.04-0.44)	0.53 (0.25-1.14)
Solid tumor without metastasis	7.05 (1.29-38.58)	0.48 (0.18-1.27)
Billing data		
*ICD-10-CM* code		
CIED infection	31.79 (5.59-180.89)	14.33 (6.1-33.65)
SSI	7.28 (0.94-56.35)	11.71 (3.64-37.64)
Pharmacy data		
No antibiotics ≥3 d	1 [Reference]	1 [Reference]
Antibiotics ≥3 d to treat *Staphylococci*	3.27 (1.08-9.93)	3.03 (1.49-6.15)
Antibiotics ≥3 d not related to *Staphylococci*	0.4 (0.03-4.79)	4.67 (0.7-31.1)
Laboratory data		
Microbiology order—cardiac	11.08 (2.84-43.14)	2.33 (1.12-4.83)
Text note data		
Infection		
Diagnosis	28.59 (3.06-267.09)	14.62 (4.44-48.08)
History	0.04 (0.01-0.22)	0.03 (0.01-0.09)
Model C statistic	0.964	0.915

Abbreviations: CIED, cardiovascular implantable electronic device; *ICD-10-CM*, *International Statistical Classification of Diseases, Tenth Revision, Clinical Modification*; OR, odds ratio; SSI, surgical site infections.

The final algorithm logistic regression model for the development sample had a C statistic of 0.96 and the AUC was 0.93. We observed that most CIED infections (41 of 47 [87.2%]) occurred in cases with a predicted probability of infection of 10% or more and determined this cutoff as a threshold for quality improvement purposes if the tool were put into practice assessing validation of the algorithm (Table 3). In the 299 cases (3.1%) with a predicted probability of infection over this threshold, PPV was 41.4% (95% CI, 31.6%-51.8%) and sensitivity and specificity were 87.2% (95% CI, 74.3%-95.2%) and 82.6% (95% CI, 78.1%-86.5%), respectively. Of the 6 true infections assigned a probability of infection less than 10%, all had the history of infection flag.

**Table 3.** Distribution of CIED Procedures by Predicted Probability of Infection and Positive Predictive Validity Results

	FY 2016-2017 development cases, No. (%) (N = 9606)	Medical record review of development sample (n = 381)			FY 2016-2017 validation cases, No. (%) (n = 9606)	Medical record review of validation sample (n = 363)		
		No.		PPV, %		No.		PPV, %
		Medical records reviewed	True infections			Medical records reviewed	True infections	
Probability range								
0-<1%	8883 (92.5)	188	1	0.5	8883 (92.5)	68	2	2.9
1%-<3%	226 (2.4)	52	2	3.8	224 (2.3)	11	1	9.1
3%-<10%	221 (2.3)	42	3	7.1	200 (2.1)	52	3	5.8
10%-<50%	176 (1.8)	60	13	21.7	193 (2)	126	36	28.6
50%-100%	100 (1)	39	28	71.8	106 (1.1)	106	65	61.3

Abbreviations: CIED, cardiac implantable electronic device; FY, fiscal year; PPV, positive predictive validity.

### Algorithm Validation

We applied the logistic regression model coefficients from the development stage to our validation data set of 9606 CIED cases and identified 299 cases with a predicted probability of an infection of 10% or more (3.1% of validation data set). Of these, 232 (77.6%) were manually reviewed, and 101 true infections were identified, yielding a PPV of 43.5% (95% CI, 37.1%-50.2%). Of the 9307 CIED cases below our 10% threshold, we reviewed 131 (1.4%) and identified 6 infections. Again, these cases were flagged has having a preprocedure infection. Algorithm sensitivity and specificity in the validation data set were 94.4% (95% CI, 88.2%-97.9%) and 48.8% (95% CI, 42.6%-55.1%), respectively. The validation logistic regression model had similar results to the final development model with a C statistic of 0.92 and an AUC of 0.90. The Figure shows the overlap between ROC in the development and validation samples.

**Figure.**
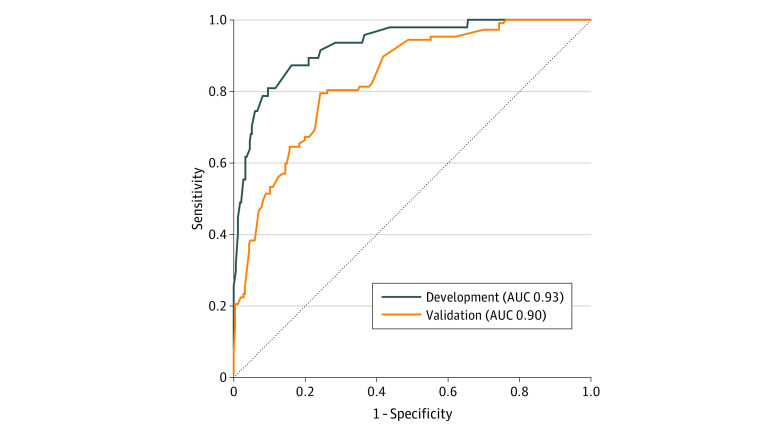
Surveillance Model Performance in Development and Validation Samples—Receiver Operating Characteristic Curves for Model Fit Development sample was 381 of 9606 cardiac implantable electronic device cases in fiscal years 2016 to 2017; validation samples 363 of 9606 cardiac implantable electronic device cases in fiscal years 2016 to 2017. AUC indicates area under curve.

### CIED Infections

Among all 19 212 CIED procedures, we reviewed 744 cases (3.9%) and identified 154 (0.8%) true procedure-related infections. We also found 138 cases (0.7%) in which an infection was present at the time of the procedure and a second procedure was being performed for infection management. Four patients developed a new infection after a revision procedure. True cases included 128 pocket infections (83.1%), 23 endocarditis and/or lead infections (14.9%), and 3 cellulitis cases (Table 4). The mean (SD) time to infection for endocarditis was 39.8 (22.7) days; pocket infections and cellulitis occurred earlier (34.8 [20.7] and 33.0 [17.5] days, respectively).

**Table 4.** Cardiac Implantable Electronic Device Infection Characteristics Based on Medical Record Review of Algorithm Development and Validation Samples From FY 2016-2017

Characteristic	Medical record review results, No. (%) (N = 154)
Type of infection	
Pocket infection	128 (83.1)
Endocarditis	23 (14.9)
Cellulitis only	3 (1.9)
History	
Infection, present on admission	13 (8.4)
History of infection	4 (2.6)
Time to infection, mean (SD), d	
Pocket infection	34.8 (20.7)
Endocarditis	39.8 (21.66)
Cellulitis only	33.0 (17.5)

Abbreviation: FY, fiscal year.

## Discussion

Infection prevention programs outside of inpatient care settings are limited even though most surgical and nonsurgical procedures occur in outpatient settings. Thus, there is a broad opportunity to reduce HAIs by focusing more attention on invasive outpatient procedural care. We developed a novel method using a combination of structured data and keyword searches of unstructured clinical text data to flag potential infections in cardiac device procedures. This new method demonstrated strong predictive value for measuring true infections and can be used as a model system for expanding HAI surveillance activities to currently uncovered areas with limited dedicated resources.

In this study, we chose the cardiac electrophysiology laboratory as the procedural setting to test this novel surveillance method for several reasons. First and foremost, cardiac device infections are highly morbid and costly; management typically requires extraction of the device with a second invasive surgery and/or open heart surgery and, as our data suggests, patients with a history of an infection are at greatly increased risk of a second infection, making infection prevention strategies even more important for infection prevention strategies. Second, formative evaluations suggest that electrophysiologists view cardiac device infections as severe and important and desire a way to measure and track them; however, resources are too limited.^24^ Third, electrophysiology laboratories represent a step away from the inpatient setting—these procedures are increasingly performed on an entirely outpatient basis—but event rates are substantial and consequences severe enough that infection prevention and measurement is an important goal.^38,39^

HAIs, including CIED infections, are difficult to detect outside of inpatient settings because they are relatively rare, often occur weeks to months after the procedure, and are not routinely recorded in easily mined EMR structured data, such as billing codes. Boggan et al^25^ examined CIED infection surveillance techniques with *ICD-9* codes and microbiology orders and their findings mirrored ours; *ICD* coding alone is an unreliable tool for detecting true infections. The VA has a cardiac procedure tracking program, and manual review of a subset of electrophysiology procedures to detect CIED infection yielded a PPV of 5%.^32^ In our previous work using an algorithm based on structured clinical data from the EMR, PPV ranged from 30% to 40%; however, this study was based on a small sample size.^26^ In the present study, we incorporated text mining strategies to discriminate procedure-related infection from other infection diagnoses in a national VA sample, and our algorithm had a PPV of 44% based on our threshold of predicted probability of infection 10% or greater. In practice, this approach was efficient—only 3% of CIED cases met our 10% threshold—and it has the potential to significantly reduce the burden of manual review for infection surveillance.

Lessons learned about informatics-based methods for HAI detection can be used to inform future studies on the expansion of surveillance to currently uncovered clinical settings and to develop similar electronic algorithms for other types of HAIs. During our development and validation, we identified several critical informatic implementation barriers that need to be considered when developing and applying a similar strategy for flagging HAIs. One challenge was the lack of utility of general antimicrobial prescriptions for identifying true infections. This was primarily due to a very high rate of antimicrobial prescriptions, most of which were for infections unrelated to the cardiac device.^26^ We found that using a limited set of antimicrobials—those most commonly used to treat *Staphylococcal* infections—was operationally useful.^40^ This finding is congruent with past studies evaluating the utility of a combination of microbiology results and antimicrobial orders for measuring methicillin-resistant *S aureus* infections.^17,41^

Another challenge was optimizing application of keyword searches to unstructured data contained in clinical notes. To address this, we focused our search on note types most likely to contain relevant information (eg, cardiology clinic notes). Another challenge with the keyword searches is a perennial problem with EMRs: serial copying of historical events. We mitigated this by including the presence of 1 of the keywords documented in clinical notes prior to the index procedure as a negative predictor. In the final model, a historical flag essentially negated future documentation of a cardiac device infection in clinical notes, so that true infections in this population required other variables (eg, *ICD-10-CM* code) to flag for their predicted probabilities to meet our 10% threshold.

### Limitations

Our study has several limitations. We conducted this study in the closed VA health care system, where the vast majority of patients (>97%) return for postoperative and postprocedural care.^41^ This was a strength for the development and validation of our algorithm because the high rate of complete data enhanced our ability to identify all true events; however, these findings may overestimate the predictive value of the algorithm in nonclosed health care settings, in which patients may receive subsequent care away from the initial health care system. This may be a particularly large limitation in rural settings, where patients may need to travel long distances to a tertiary care facility that performs CIED procedures but then return to a local facility with fewer services for subsequent care. Second, because we reviewed only a sample of cases, it is possible that our algorithm has lower predictive value than is estimated based on our methods. However, this was mitigated by our use of separate development and validation samples to ensure algorithm accuracy and predictive value. Furthermore, event rates predicted by our algorithm are similar to event rates in other studies,^42^ which reassures us that the algorithm is appropriately flagging high-probability cases. Finally, it is possible that use of advanced informatic methods such as machine learning may have improved algorithm performance; however, our logistic regression model based on a relatively small number of cases with medical record review performed well within the VA setting and required significantly less training data than machine learning analyses.^43^

## Conclusions

The evolution of infection prevention programs to include outpatient and procedural areas is crucial as health care delivery is increasingly provided outside of traditional settings. In this study, we successfully combined structured and text data in the VA EMR to accurately and efficiently flag nonsurgical HAIs. Our method of algorithm development and validation using EMR-derived data, including clinicians’ notes to triage manual medical record review, has broad application beyond CIED infections. Furthermore, as integrated health care systems use EMRs in more outpatient settings and expand the use of CDWs, this approach to HAI surveillance can be replicated in non-VA care to enhance and support quality measurement.

## Notes

Supplement. **eTable 1.** CIED Infections by Comorbidity **eTable 2.** Definition of Cardiac Implantable Electronic Device (CIED) Infection **eTable 3.** List of *ICD-10-CM* Infection Codes **eTable 4.** List of Keywords in Text Mining
