# Supplementary material for: Novel Method to Flag Cardiac Implantable Device Infections by Integrating Text Mining With Structured Data in the Veterans Health Administration’s Electronic Medical Record
Source: JAMA Netw Open. 2020 Sep 21;3(9):e2012264. doi: 10.1001/jamanetworkopen.2020.12264 (PMC7506515; doi:10.1001/jamanetworkopen.2020.12264)
Supplement: Supplement. — eTable 1. CIED Infections by Comorbidity eTable 2. Definition of Cardiac Implantable Electronic Device (CIED) Infection eTable 3. List of ICD-10-CM Infection Codes eTable 4. List of Keywords in Text Mining [file jamanetwopen-e2012264-s001.pdf]

Supplementary Online Content

Mull HJ, Stolzmann KL, Shin MH, Kalver E, Schweizer ML, Branch-Elliman W. Novel method to flag cardiac implantable device infections by integrating text mining with structured data in the Veterans Health Administration's electronic medical record. *JAMA Netw Open*. 2020;3(9):e2012264. doi:10.1001/jamanetworkopen.2020.12264

eTable 1. CIED Infections by Comorbidity

eTable 2. Definition of Cardiac Implantable Electronic Device (CIED) Infection

eTable 3. List of *ICD-10-CM* Infection Codes

eTable 4. List of Keywords in Text Mining

This supplementary material has been provided by the authors to give readers additional information about their work.

© 2020 Mull HJ et al. *JAMA Network Open.*

eTable 1. CIED Infections by Comorbidity

Comorbidities	Flag Rate in FY16-17 Development Sample (n=9,606)	Total Reviewed (n=381)	With Infection (n=47)
AIDS	54 (0.56%)	2 (0.52%)	0
Alcohol abuse	713 (7.42%)	42 (11.02%)	6 (12.77%)
Deficiency Anemias	2,005 (20.87%)	120 (31.5%)	11 (23.4%)
Rheumatoid arthritis/ collagen vascular disorder	180 (1.87%)	6 (1.57%)	1 (2.13%)
Chronic blood loss anemia	126 (1.31%)	7 (1.84%)	0
Congestive heart failure	4,872 (50.72%)	230 (60.37%)	22 (46.81%)
Chronic pulmonary disease	2,725 (28.37%)	132 (34.65%)	15 (31.91%)
Coagulopathy	548 (5.7%)	38 (9.97%)	8 (17.02%)
Depression	1,759 (18.31%)	84 (22.05%)	11 (23.4%)
Diabetes w/o chronic complications	3,870 (40.29%)	187 (49.08%)	23 (48.94%)
Diabetes w/ chronic complications	2,661 (27.7%)	146 (38.32%)	17 (36.17%)
Drug abuse	359 (3.74%)	23 (6.04%)	1 (2.13%)
Hypertension	7,855 (81.77%)	321 (84.25%)	41 (87.23%)
Hypothyroidism	1,169 (12.17%)	58 (15.22%)	6 (12.77%)
Liver disease	578 (6.02%)	32 (8.4%)	4 (8.51%)
Lymphoma	109 (1.13%)	5 (1.31%)	1 (2.13%)
Fluid and electrolyte disorders	1,822 (18.97%)	110 (28.87%)	12 (25.53%)
Metastatic cancer	81 (0.84%)	4 (1.05%)	1 (2.13%)
Other neurological disorders	964 (10.04%)	54 (14.17%)	7 (14.89%)
Obesity	1,991 (20.73%)	102 (26.77%)	10 (21.28%)
Paralysis	231 (2.4%)	15 (3.94%)	3 (6.38%)
Peripheral vascular disease	1,582 (16.47%)	100 (26.25%)	14 (29.79%)
Psychoses	634 (6.6%)	33 (8.66%)	6 (12.77%)
Pulmonary circulation disease	412 (4.29%)	25 (6.56%)	3 (6.38%)
Renal failure	2,463 (25.64%)	123 (32.28%)	14 (29.79%)
Solid tumor without metastasis	792 (8.24%)	34 (8.92%)	7 (14.89%)
Peptic ulcer disease with bleeding	78 (0.81%)	5 (1.31%)	1 (2.13%)
Valvular disease	1,788 (18.61%)	111 (29.13%)	13 (27.66%)

© 2020 Mull HJ et al. *JAMA Network Open.*

Weight loss	493 (5.13%)	36 (9.45%)	4 (8.51%)

© 2020 Mull HJ et al. *JAMA Network Open.*

eTable 2. Definition of Cardiac Implantable Electronic Device (CIED) Infection

Elements Confirming CIED Infection	Elements Ruling Out CIED Infection
1) Occurs within 90 days after the CIED<br><br>**AND**<br><br>2) Clinician diagnosis of infection (pocket, endocarditis, leads)<br><br>**OR**<br><br>3) Patient has at least *one* of the following:<br><br>*Pocket infection:*<br><br>• Erythema (swelling) OR drainage from pocket AND treatment (surgical or antibiotics)<br>• Erythema/drainage/pus from pocket AND positive laboratory confirmation of microorganisms<br>• Pus from pocket AND treatment (surgical or antibiotics)<br>• Fever AND purulent drainage/erythema from pocket (with or without antibiotics, microorganisms, elevated white blood cell count)<br>• Abscess at pocket site<br><br>*Endocarditis:*<br><br>• Positive blood cultures AND clinical diagnosis of permanent pacemaker infection<br>• Positive blood cultures AND echo/other imaging with evidence of lead involvement<br>• Satisfies Duke's criteria[1]<br><br>*Superficial cellulitis:*<br><br>• Warmth, redness or erythema at the site with or without fever with physician treatment AT THE SITE of the cardiac device implant	*Do not report the following conditions as CIED infection:*<br><br>• Stitch abscess (minimal inflammation and discharge confined to the points of suture penetration).<br>• Cellulitis in other locations than incision site.<br><br>*Do not record a CIED infection if the CIED procedure was performed to address an existing infection.*

NOTES: Our definition of CIED infection was based on the National Healthcare Safety Network's invasive procedure surgical site infection definition and definitions recommended by

© 2020 Mull HJ et al. *JAMA Network Open.*

national and international societies.[2-4] Based on the combined recommendations of these guidelines, infection status was determined based on physician documentation of “CIED infection” or presence of symptoms, laboratory tests and/or antibiotic treatment up to 90 days post-procedure. CIED infections present at the time of procedure (sometimes CIED procedures are performed to correct infections from prior procedures), superficial cellulitis and stitch abscesses were excluded from the definition of procedure-related infection, in line with NSHN reporting recommendations.[5]

References:

1. Durack DT, Lukes AS, Bright DK. New criteria for diagnosis of infective endocarditis: utilization of specific echocardiographic findings. Duke Endocarditis Service. *Am J Med.* 1994;96(3):200-209.
2. Baddour LM, Epstein AE, Erickson CC, et al. Update on Cardiovascular Implantable Electronic Device Infections and Their Management. *Circulation.* 2010;121(3):458-477.
3. Rahman R, Saba S, Bazaz R, et al. Infection and readmission rate of cardiac implantable electronic device insertions: An observational single center study. *American journal of infection control.* 2016;44(3):278-282.
4. Sandoe JAT, Tayebjee MH, Howard P, et al. Guidelines for the diagnosis, prevention and management of implantable cardiac electronic device infection. Report of a joint Working Party project on behalf of the British Society for Antimicrobial Chemotherapy (BSAC, host organization), British Heart Rhythm Society (BHRS), British Cardiovascular Society (BCS), British Heart Valve Society (BHVS) and British Society for Echocardiography (BSE). *Journal of Antimicrobial Chemotherapy.* 2014;70(2):325-359.
5. CDC. Surgical Site Infection (SSI) Event. http://www.cdc.gov/nhsn/PDFs/pscManual/9pscSSIcurrent.pdf. Published 2015. Updated January 2016. Accessed April 7, 2016.

© 2020 Mull HJ et al. *JAMA Network Open.*

eTable 3. List of *ICD-10-CM* Infection Codes

Infection Code Flags	ICD-10-CM Infection Codes
CIED infection:	‘B37.6%’ Candidal endocarditis;<br>‘I33%’ Acute and subacte endocarditis;<br>‘I35%’ Aortic valve endocarditis;<br>‘I38%’ Valve unspecified endocarditis;<br>‘I39%’ Endocarditis and heart valve disorders NOS;<br>'T82.6%' Infection and inflammatory reaction due to cardiac device, implant or graft;<br>'T82.7%' Cardiac device infection
Surgical Site Infection:	'T81.3%' Disruption of wound;<br>'T81.4%' Infection following procedure
Site non-specific systemic infection:	'A41.01%' Sepsis due to MSSA;<br>'A41.02%' Sepsis due to MRSA;<br>'A49.01%' MSSA infection NOS;<br>'A49.02%' MRSA infection NOS;<br>'A49.1%' Strep infection, site unspecified;<br>‘R78.81%’ Bacteremia;<br>'B95%' Strep, staph, and enterococcus infection cause of disease

NOTE: Timeframe: up to 90 days post-procedure. CIED= cardiac implantable electronic device; NOS= not otherwise specified

© 2020 Mull HJ et al. *JAMA Network Open.*

eTable 4. List of Keywords in Text Mining

Type of Search	Final Keyword List	Tested but Rejected Keywords
**Text Chart Keywords (from selected clinical note types)**<br><br>Timeframe: from 3 days post procedure up to 90 days post procedure	“Device” & “infection”<br>“Pacemaker” & “infection”<br>“CIED & “infection”<br>“Defibrillator” & “infection”<br>“ICD” & “infection”<br>“AICD” & “infection”<br>“PM” & “infection”<br>“PMM” & “infection”<br>“Generator” & “infection”<br>“Pocket” & “infection”<br>“Wound” & “abscess”<br>“Pocket & “abscess”<br>“Stitch” & “abscess”<br>"Endocarditis"<br>“Lead” & “infection”<br>“Device” & “erosion”	“Wound” & “cellulitis”<br>“Superficial & “abscess”<br>"Cellulitis around incision"<br>"Cellulitis at incision site"<br>“Superficial” & “cellulitis”<br>“Dehiscence”<br>“Poor wound healing”<br>“Open” & “wound”<br>“Open” & “incision”<br>“Pocket” & “tenderness”<br>“Pocket” & “warmth”<br>“Pocket” & “erythema”<br>“Pocket” & “redness”<br>“Lead” & “vegetation”<br>“Valve” & “vegetation”<br>“Vegetation” & “valve”<br>“Valve” & “endocarditis”<br>“Lead” & “involvement”<br>“Mobile” & “mass”<br>“Abscess”<br>“MRSA”<br>“MSSA”<br>“S. aureus”<br>“Staph aureus”<br>“Staph non-aureus”

© 2020 Mull HJ et al. *JAMA Network Open.*

***Staphylococci* Flag:**<br><br>**Organism Keywords (among positive micro results)**<br><br>Timeframe: up to 90 days post-procedure	=1 if Organism is 'Staph Aureus" defined: like %staph% and organism like: "%aureus%" or "%mrsa%" or "%coagulase positive%"<br><br>=2 if Organism is 'Non-Aureus Staph" for all other non-aureus %staph% species

NOTE: CIED= cardiac implantable electronic device

© 2020 Mull HJ et al. *JAMA Network Open.*
